# Cytoplasmic HuR Expression Enhances Chemoresistance in Pleural Mesothelioma Through Increased Expression of CALB2, Promotion of the E2F Pathway, and Suppression of the p53 Pathway

**DOI:** 10.1111/1759-7714.70062

**Published:** 2025-04-09

**Authors:** Susumu Kirimura, Morito Kurata, Hironori Ishibashi, Yusuke Taniguchi, Yuko Kinowaki, Keisuke Sugita, Kenichi Okubo

**Affiliations:** ^1^ Division of Pathology Institute of Science Tokyo Hospital Tokyo Japan; ^2^ Department of Comprehensive Pathology, Graduate School of Medical and Dental Sciences Institute of Science Tokyo Tokyo Japan; ^3^ Department of Thoracic Surgery, Graduate School of Medical and Dental Sciences Institute of Science Tokyo Tokyo Japan; ^4^ Department of Pathology The Cancer Institute Hospital, Japanese Foundation for Cancer Research Tokyo Japan

**Keywords:** chemoresistance, human antigen R (HuR), mesothelioma

## Abstract

**Introduction:**

Chemotherapy is crucial for treating pleural mesothelioma; however, the outcomes are poor, necessitating an urgent need to study the mechanism of chemotherapy resistance in mesothelioma cells. Human antigen R (HuR), an RNA‐binding protein and key post‐transcriptional regulator of mRNA, is linked to poor prognosis in cancers like mesothelioma. We investigated the involvement of cytoplasmic HuR expression in drug resistance mechanisms in mesothelioma.

**Methods:**

We retrospectively evaluated cytoplasmic HuR expression in 30 patients with pleural mesothelioma who underwent surgical resection using immunohistochemistry. We also examined the role of forced cytoplasmic expression of HuR in drug resistance using mesothelioma cell lines and performed RNA‐Seq analysis to identify gene expression changes responsible for drug resistance acquisition via HuR cytoplasmic expression.

**Results:**

Patients with mesotheliomas who expressed cytoplasmic HuR exhibited significantly worse disease‐free survival following post‐operative chemotherapy. Forced cytoplasmic HuR expression in mesothelioma cell lines increased chemotherapy resistance through increased expression of *CALB2*, upregulation of the E2F pathway and suppression of the p53 pathway.

**Conclusions:**

Cytoplasmic HuR expression increases the chemoresistance and postoperative recurrence risk of pleural mesothelioma, making it a potential biomarker for predicting therapeutic prognosis. However, the mechanism of HuR transfer to the cytoplasm remains unclear for therapeutic application.

## Graphical abstract

We found that cytoplasmic HuR expression increases chemoresistance and postoperative risk of recurrence in pleural mesothelioma. It has been suggested that this is due to increased expression of CALB2, up‐regulation of the E2F pathway and suppression of the p53 pathway.

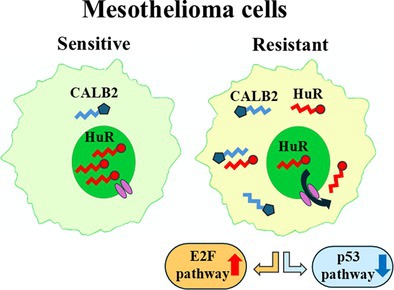

## 1 Introduction

Diffuse malignant mesothelioma of the pleura is a rare, aggressive tumour primarily linked to asbestos exposure, with an age‐standardised incidence rate of approximately 0.30/100 000 persons and poor prognosis [1]. Chemotherapy is the mainstay of treatment in unresectable cases of mesothelioma. Even in resectable cases, surgery alone is challenging, and multimodal therapy combining chemotherapy, radiation therapy and immune checkpoint inhibitors has been reported [2]. However, even with the main regimen of cisplatin (CDDP) and pemetrexed (PEM) combination therapy, the response rate is only 32.5%, and chemotherapy alone has a median survival of 7.6 months [3]. Therefore, there is an urgent need to elucidate the mechanism of chemotherapeutic resistance in mesothelioma cells, which presumably involve stemness‐related pathways, splicing deregulation, microRNA and Notch pathway aberrations [4, 5], though the details remain unclear.

Human antigen R (HuR), also known as embryonic lethal abnormal visual‐like protein, is an RNA‐binding protein ubiquitously expressed in all human tissues and is a critical post‐transcriptional regulator of gene expression that influences RNA localisation, stability and translation [6, 7]. In the nucleus, HuR binds to pre‐mRNA intronic sequences of target mRNAs and is involved in splicing, stability and alternative polyadenylation [8, 9]. On the other hand, when HuR translocates to the cytoplasm, it stabilises and enhances the expression of its target mRNA by binding to AU‐rich elements in their 3' untranslated regions [10, 11]. The cytoplasmic expression of HuR has been implicated in the progression of various cancers and is associated with poor prognosis [7], including mesothelioma [12]. Since HuR's involvement in drug resistance has been reported in various cancers, with no specific evidence in mesothelioma [13], we investigated the possibility and mechanism of cytoplasmic HuR (cHuR) contributing to drug resistance in mesothelioma.

## 2 Methods

### 2.1 Patients and Pathological Specimens

Pathological specimens were collected retrospectively from 30 patients who underwent surgical resection for pleural mesothelioma at the Tokyo Medical and Dental University (TMDU) Hospital between 2010 and 2017. The specimens were routinely fixed in 10% neutralised formalin and embedded in paraffin for conventional histopathological examination. Patients who received preoperative chemotherapy were excluded from the study.

### 2.2 Immunohistochemistry

Formalin‐fixed paraffin‐embedded (FFPE) tissues were sliced to 4 μm thickness. Heat‐based antigen retrieval was performed by incubating the specimens at 95°C for 20 min in citrate buffer (pH 6.0), followed by endogenous peroxidase blocking with 3% hydrogen peroxide and normal horse serum blocking (ABC Kit; Vector Laboratories, Burlingame, CA, USA). Specimens treated with primary antibody (mouse monoclonal anti‐HuR, 1:100 dilution; clone number sc5261; Santa Cruz Biotechnology, Dallas, TX, USA) were incubated overnight at 4°C. Antibody detection was performed using an ABC Kit (Vector Laboratories) by staining the slides with diaminobenzidine (DAB; Vector Laboratories) and counterstaining with haematoxylin.

### 2.3 Cell Lines and Culture

The mesothelioma cell line, ACC‐MESO1 (MESO1), was procured from the RIKEN BioResource Center (Tsukuba, Japan) [14]. The cells were cultured in Roswell Park Memorial Institute (RPMI)‐1640 medium containing L‐glutamine and phenol red (Wako Pure Chemical Industries Ltd., Osaka, Japan) in the presence of 10% foetal bovine serum and 1% penicillin–streptomycin with 5% CO_2_ at 37°C.

### 2.4 Establishment of a cHuR‐Expressing ACC‐MESO1 Cell Line Using Recombinant Lentivirus and Lentiviral Transduction

Constructs of consecutive nuclear export signal (NES) or nuclear localization signal (NLS) sequences and *HuR* gene sequences were synthesized as gBlock gene fragments. The fragments were transferred to an entry vector using the Invitrogen Gateway BP Clonase reaction (Thermo Fisher Scientific) and then transferred into the destination vector using the Invitrogen Gateway LR Clonase reaction (Thermo Fisher Scientific) to obtain the recombinant plasmids carrying NES‐HuR and NLS‐HuR. A schema of these recombinant plasmids is shown in Figure S1. Recombinant lentiviral particles were obtained by co‐transfection of packaging plasmids into HEK293T cells.

### 2.5 Immunofluorescence Microscopy for Subcellular Localization of HuR

ACC‐MESO1 cells (5 × 10^3^) transfected with NES‐HuR and NLS‐HuR were cultured on Falcon 4‐well Culture Slides (BD Falcon, NJ, USA). Cells were fixed in 100% ethanol at −20°C for 20 min, followed by incubation overnight with a mouse monoclonal anti‐HuR antibody (Santa Cruz) at a 1:100 dilution at 4°C. The cells were then stained with a fluorescein isothiocyanate‐conjugated anti‐mouse antibody (Dako Cytomation, Glostrup, Denmark) at a 1:100 dilution for 20 min at 20°C. The slides were mounted with mounting medium (Dako Cytomation) containing 4',6‐diamidino‐2‐phenylindole (DAPI; Abbott Molecular Inc., Des Plaines, IL, USA), and images were captured using a BZ‐X810 fluorescence microscope (KYENCE, Tokyo, Japan).

### 2.6 Cell Viability Assessment Following Drug Exposure Using the Dimethylthiazol‐Carboxymethoxyphenyl‐Sulfophenyl‐Tetrazolium (MTS) Assay

The cells were seeded at 1 × 10^3^ cells/well in a 96‐well culture plate containing 200 μL media/well and treated with serially diluted CDDP or PEM. After 5 days of treatment with CDDP or PEM, cell viability was evaluated by MTS assay (Promega, Madison, WI, USA), following the manufacturer's protocol.

### 2.7 Assessment of Propidium Iodide Staining

The cells were seeded at a density of 4 × 10^3^ cells/well in a 24‐well culture plate containing 400 μL media/well and treated with either 10 μM CDDP or 50 μM PEM for 5 days, following which the medium was discarded and the cells were washed with phosphate‐buffered saline. Propidium iodide PI (Sigma‐Aldrich, St. Louis, MO, USA) was added to each well, and the percentage of PI‐positive cells was evaluated using a BD FACSCanto II analyser (Becton Dickinson and Company, Franklin Lakes, NJ, USA).

### 2.8 RNA Sequencing Analysis

Total RNA was extracted from NES‐HuR transgenic ACC‐MESO1 cells and non‐target cells using the RNeasy Mini Kit (QIAGEN, Hilden, Germany). RNA sequencing was performed by Novogene in Beijing, China, using a NovaSeq 6000 sequencing system (Illumina, San Diego, CA, USA). Volcano plots were generated using the Novosmart software (Novogene). RNA expression data were analysed and visualised using Gene Set Enrichment Analysis (GSEA) software (version 4.3.2, https://www.gsea‐msigdb.org/gsea/index.jsp).

### 2.9 Western Blotting

Cells (1 × 10^6^) were lysed in a sodium dodecyl sulphate (SDS) buffer. Samples were separated by SDS‐polyacrylamide gel electrophoresis (PAGE) (Bio‐Rad, Hercules, CA, USA) and electrotransferred onto Immobilon polyvinylidene difluoride (PVDF) membranes (Millipore, Burlington, MA, USA). The membranes were incubated in Bullet Blocking One (Nacalai Tesque, Kyoto, Japan) for 5 min at 20°C. The membranes were then incubated with the primary antibodies, anti‐calretinin (Santa Cruz, sc‐365956, 1:100), anti‐PIM1 (Santa Cruz, sc‐13513, 1:100), anti‐β‐actin (Cell Signaling Technology, #4967, 1:1000), anti‐E2F1 (Santa Cruz, sc‐251, 1:100) or anti‐p53(Leica Biosystems, Newcastle, United Kingdom, DO‐7, 1:200), overnight at 4°C. The next day, the membranes were incubated with horseradish peroxidase‐conjugated secondary antibodies of the animal species corresponding to each primary antibody at a 1:5000 dilution for 1 h at 20°C. Protein bands were visualised using Clarity Western ECL Substrate (Bio‐Rad) and quantified by densitometry using the ImageJ software (National Institutes of Health, USA).

### 2.10 Statistical Analyses

Correlations between the two groups were assessed using Fisher's exact test. Kaplan‐Meier survival curves were used to estimate overall survival (OS) and disease‐free survival (DFS) rates. The log‐rank test was used to analyse the differences in OS and DFS between groups. Univariate and multivariate analyses were performed using a Cox proportional hazards regression model. The Mann‐Whitney *U*‐test was used to compare the two groups, and one‐way analysis of variance (ANOVA) with Dunnett's or Bonferroni's test was used to compare multiple groups. The results were obtained independently in triplicate and are presented as mean ± standard deviation (SD). Statistical significance was set at *p* < 0.05. For GSEA, results were considered significant when nominal (NOM)‐*p* < 0.05 and false discovery rate (FDR)‐*q* < 0.25 were met. All statistical analyses were performed using the GraphPad Prism 9 software (https://www.graphpad.com/scientific‐software/prism/www.graphpad.com/scientific‐software/prism/).

## 3 Results

### 3.1 HuR Is Expressed in the Cytoplasm in Half of All Patients With Mesothelioma

**FIGURE 1:**
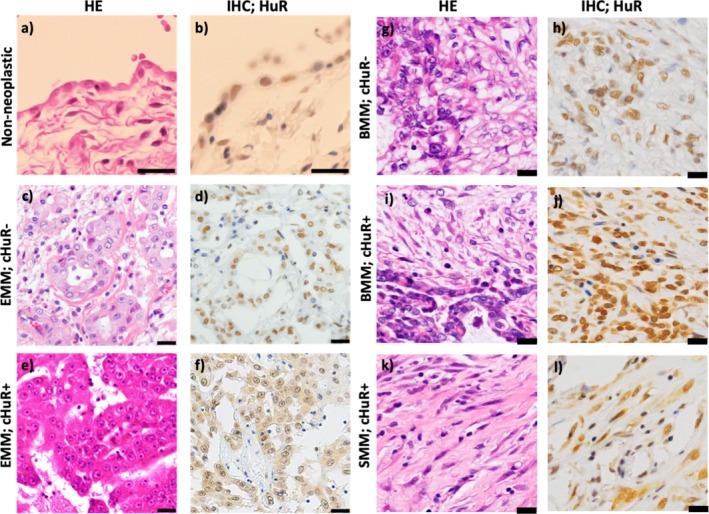
HuR immunostaining. (a, b) In the control case, HuR is positive in the nuclei but negative in the cytoplasm. (c, d) Negative cHuR in some epithelioid mesothelioma cases. (e, f) Positive cHuR in some mesothelioma cases. (g, h) Negative cHuR in a case of biphasic mesothelioma. (i, j) Positive cHuR in many biphasic mesothelioma cases. (k, l) Positive cHuR in a sarcomatoid mesothelioma case. BMM, biphasic mesothelioma; cHuR, cytoplasmic HuR; EMM, epithelioid mesothelioma; HuR, Human antigen R; SMM, sarcomatoid mesothelioma. Scale bars = 10 μm.

Immunostaining performed to determine the expression status of HuR in mesothelioma cells showed (Figure 1) positive HuR staining in the nucleus of the non‐neoplastic mesothelial cells, but negative in the cytoplasm. Out of the 30 patients with mesothelioma, 50.0% showed positive HuR staining in the cytoplasm, whereas the rest of the samples showed positive staining in the nucleus. Table 1 summarises the clinical characteristics of the patients with mesothelioma in this study. There were no significant differences between the cHuR‐positive and ‐negative groups in terms of clinicopathological factors such as age, sex, asbestos exposure, histology, pathological tumour, lymph nodes, metastasis (TMN) stage; and post‐operative chemotherapy.‐

**TABLE 1:** Patient characteristics.

Characteristics	Variable	cHuR		*p*
		Positive	Negative	
Age	Median (range)	69 (48–76)	65 (50–75)	0.6156
Sex	Male	11	15	0.0996
	Female	4	0	
Asbestos exposure	(+)	12	13	1.0000
	(−)	3	2	
Histology	Epithelioid	11	13	0.6513
	Biphasic+ sarcomatous	4	2	
pStage	≧ IB	13	10	0.3898
	IA	2	5	
Chemotherapy	(+)	8	12	0.2451
	(−)	7	3	

*Note: p* value was calculated using the Mann‐Whitney *U* test or Chi‐Square test, where appropriate.

Abbreviations: cHuR, cytoplasmic HuR; pStage, pathological Stage.

### 3.2 CHuR‐Positive Groups Showed a Poor Prognosis in DFS Following Postoperative Chemotherapy Administration

**FIGURE 2:**
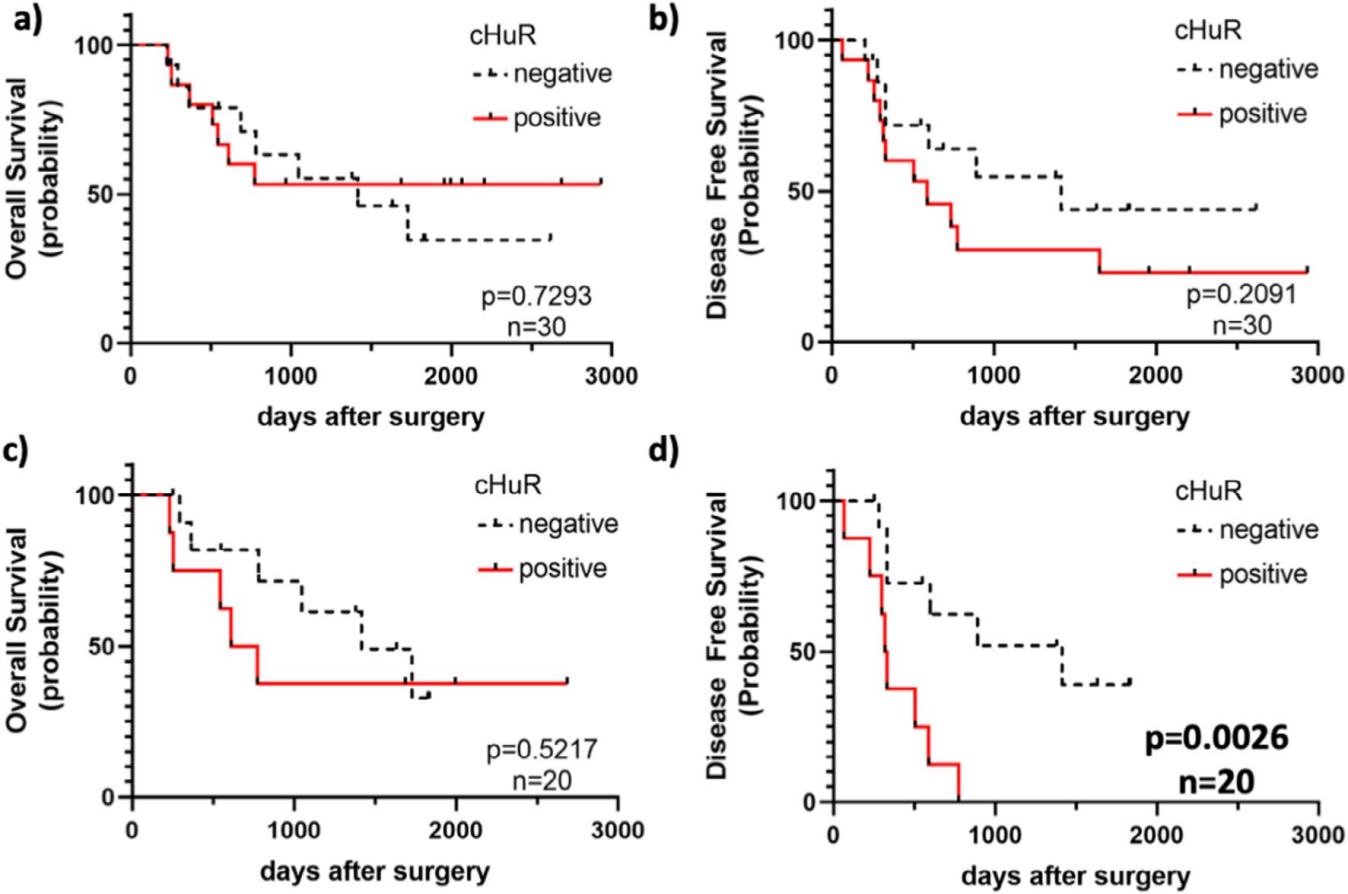
Kaplan‐Meier analysis. (a) Overall survival (OS) and (b) disease‐free survival (DFS) in all mesothelioma patients. (c) OS and (d) DFS in patients with mesothelioma with postoperative chemotherapy. (d) When limited to patients who received postoperative chemotherapy, DFS was significantly shorter in the cHuR‐positive groups. cHuR, cytoplasmic HuR; HuR, Human antigen R.

We examined the correlation between cHuR expression and patient prognosis (Figure 2). The log‐rank test revealed no significant differences in OS or DFS between the cHuR‐positive and ‐negative groups when all patients were considered (Figure 2a,b). However, when the analysis was limited to patients who received postoperative chemotherapy, DFS was significantly shorter in the cHuR‐positive group (Figure 2d, *p* = 0.0026). Table 2 shows the results of univariate and multivariate analyses of patients who received postoperative chemotherapy using the Cox proportional hazards model for age, sex, TNM stage, histological classification, nuclear grade (calculated according to the 2021 WHO criteria [15]), necrosis and cHuR positivity. Among these variables, necrosis and cHuR correlated with DFS in univariate analysis. Furthermore, we found that cHuR was the only independent predictor of DFS, as determined by multivariate analysis (hazard ratio, 5.1854; *p* = 0.0099).

**TABLE 2:** Univariate and multivariate cox regression analysis (*n* = 20).

Variables	Univariate	Multivariate	Hazard ratio	95% CI
	*p*	*p*		
Age (≧ 65 vs. < 65)	0.6441			
Sex (male vs. female)	0.7105			
pStage (≧ IB vs. IA)	0.0513			
Biphasic or sarcomatoid (yes vs. no)	0.0601	0.1397	3.2138	0.6827–15.1291
Nuclear grade (≧ II vs. I)	0.405			
Necrosis ((+) vs. (−))	0.0074			
cHuR (positive vs. negative)	0.0026	0.0099	5.1854	1.4884–18.1137

Abbreviations: cHuR, cytoplasmic HuR; pStage, pathological stage.

### 3.3 Alteration of Drug Susceptibility by cHuR Expression

**FIGURE 3:**
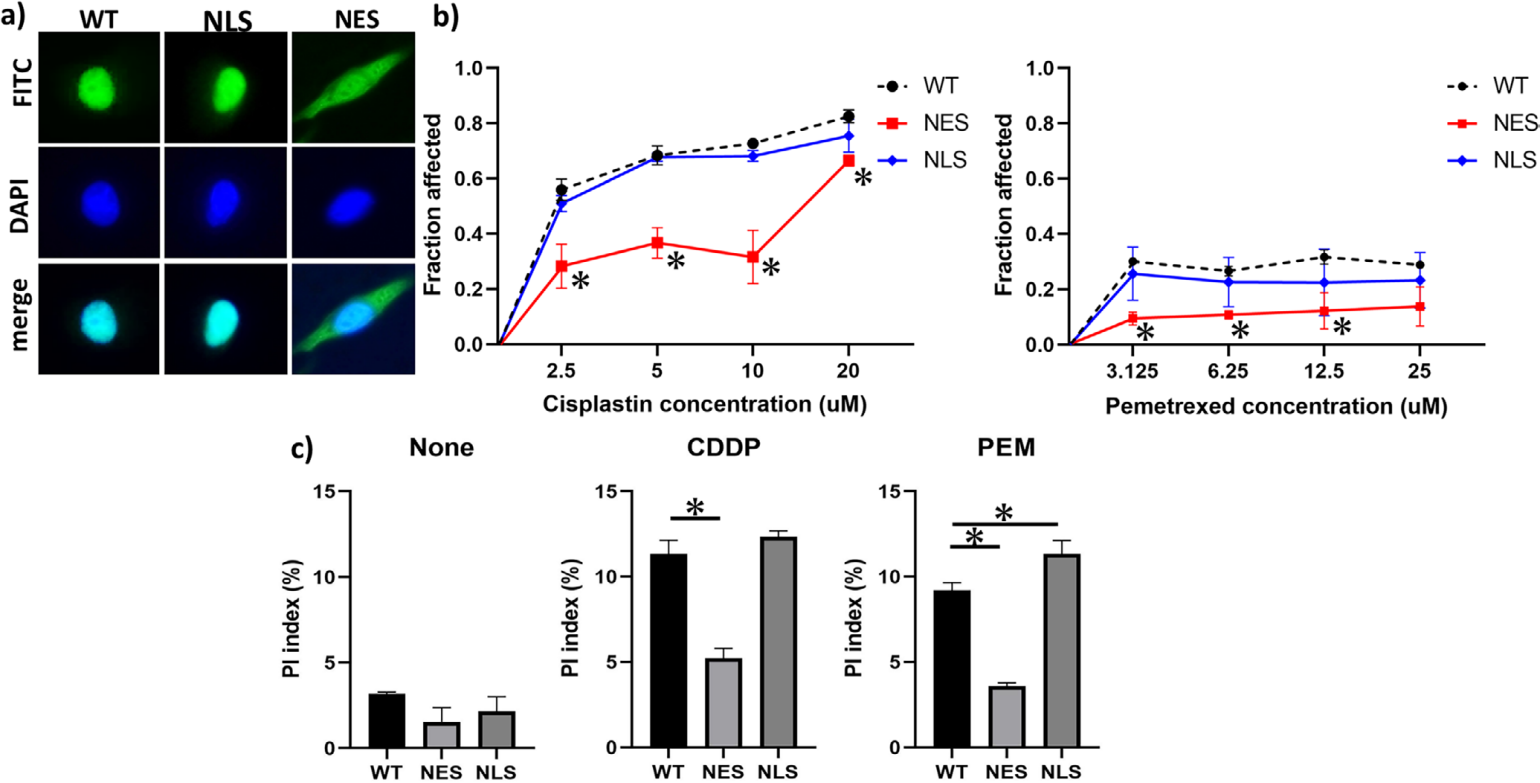
Alteration of drug sensitivity by cytoplasmic HuR expression. (a) Immunofluorescent analysis demonstrates cytoplasmic expression of HuR in the NES‐HuR transgenic cells (NES). (b) MTS assay shows a lower rate of chemotherapy‐induced decrease in proliferative activity in NES. (c) Propidium Iodide assay shows a reduced percentage of cells killed by chemotherapy in NES. CDDP, cisplatin; HuR, Human antigen R; MTS, dimethylthiazol‐carboxymethoxyphenyl‐sulfophenyl‐tetrazolium; NES, nuclear export signal; NLS, nuclear localization signal; PEM, pemetrexed; WT, wild type. *p < 0.05.

To demonstrate whether cytoplasmic expression of HuR confers drug resistance, we first attempted to express HuR in the cytoplasm of mesothelioma cells by introducing the *HuR* gene with attached NES sequences (*NES‐HuR* gene) using lentiviral vectors. As shown in Figure 3a, wild‐type mesothelioma cells and those transfected with the *HuR* gene with an attached NLS sequence (*NLS‐HuR* gene) were positive for HuR only in the cytoplasm. In contrast, mesothelioma cells transfected with NES‐HuR were positive for the nucleus and cytoplasm. The MTS assay (Figure 3b) showed that the rate of decrease in cell proliferative activity was significantly lower in mesothelioma cells transfected with the NES‐HuR gene in CDDP‐ and PEM‐treated groups. Furthermore, the percentage of dead cells labelled with PI was significantly lower in the NES‐HUR‐transduced mesothelioma cells treated with either CDDP or PEM (Figure 3c).

### 3.4 CHuR Expression Enhances mRNA Expression of Genes Involved in Drug Resistance

**FIGURE 4:**
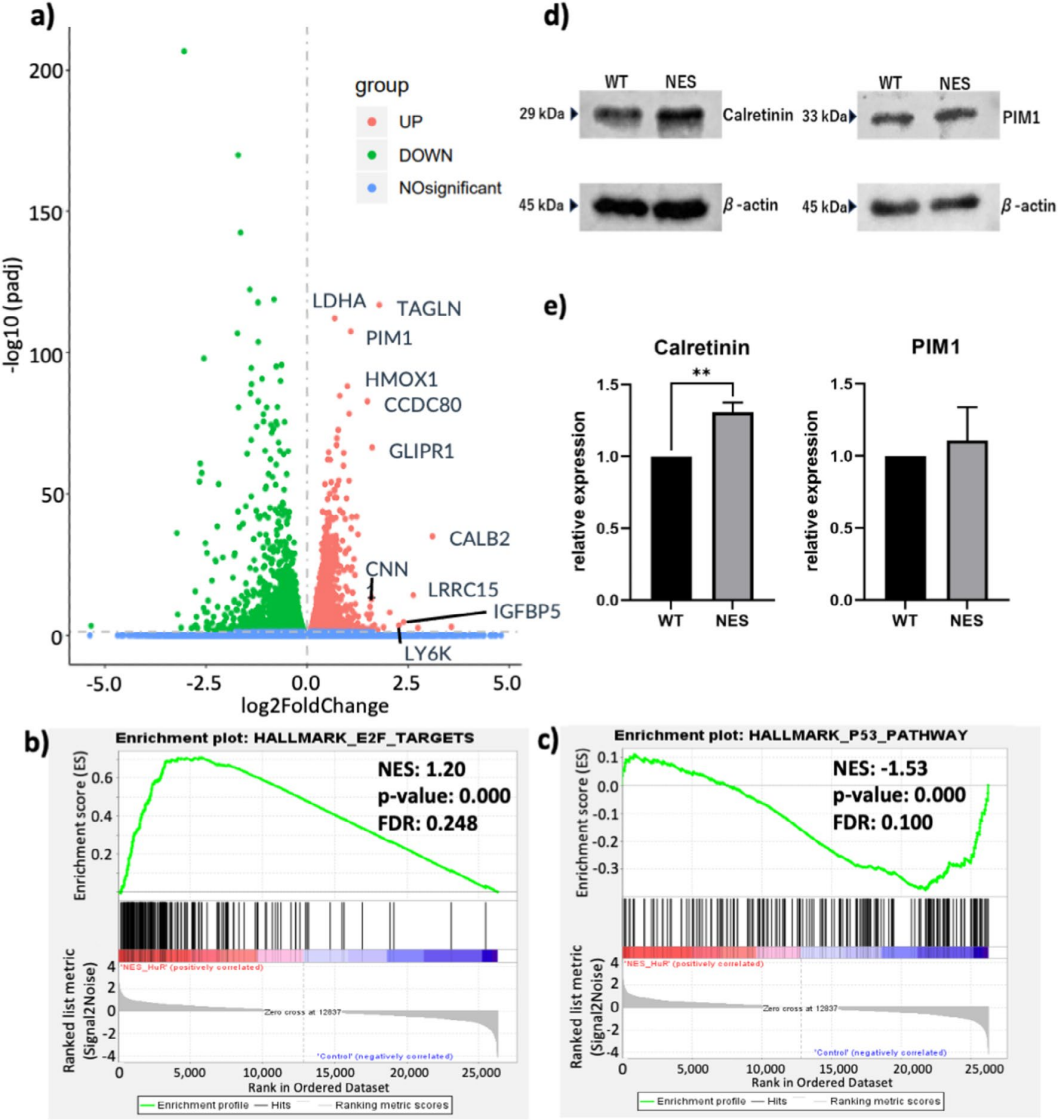
RNA‐sequencing analysis. (a) Volcano plot. Many genes involved in drug resistance were upregulated in the NES‐HuR transgenic cells (NES). (b, c) GSEA analysis: (b) The E2F target gene cluster was upregulated in NES. (c) The p53 pathway gene cluster was downregulated in NES. (d) Western blotting showed that Calretinin and PIM1 tended to be increased in NES. (e) Densitometry analysis showed a significant increase in Calretinin protein in NES. Nuclear export signal, **p < 0.05. GSEA, gene set enrichment analysis; HuR, Human antigen R; NES, nuclear export signal.

RNA‐seq analysis was used to examine changes in gene expression owing to cHuR expression. Since cHuR contributes to mRNA stabilization [10, 11], we focused mainly on upregulated mRNAs to determine the direct effects of cHuR. As shown in the volcano plot in Figure 4a, NES‐HuR‐transfected cells showed increased mRNA expression of various drug‐resistance genes, including *CALB2*, *LRRC15*, *IGFBP15*, *LY6K*, *TAGLN*, *CNN1*, *GLIPR1* and *PIM1*. GSEA showed that E2F target genes were upregulated in NES‐HuR‐transfected cells (Figure 4b and Table S1). In addition, multiple pathways, including the p53 pathway, were suppressed in the NES‐HuR‐transfected cells (Figure 4c and Table S2). For the expression of E2F and p53 itself, no clear tendency at the mRNA levels and no significant changes at the protein level were observed by forced induction of HuR (Table S5 and Figure S2).

Since *CALB2* was the most differentially expressed drug‐resistance gene observed in the RNA‐seq among those significantly upregulated in NES‐HuR‐transfected cells and PIM1, which is involved in the E2F pathway [16], western blotting was performed to confirm the differences in the protein expression of calretinin and PIM1. Figure 4d,e shows that calretinin was significantly upregulated in NES‐HuR‐transfected cells, whereas PIM1 tended to be upregulated, but the increase was not significant. Thus, we hypothesised that cytoplasmic HuR expression in mesothelioma cell lines conferred drug resistance by modifying the expression of numerous genes, including increased expression of CALB2, promoting the E2F pathway and suppressing the p53 pathway.

## 4 Discussion

In this study, we found that in patients with mesothelioma receiving post‐surgical chemotherapy, DFS was significantly shorter if they expressed cHuR, suggesting that cHuR worsens prognosis by acquiring chemoresistance. This was confirmed by the decreased sensitivity to chemotherapy in cells with forced expression of cHuR.

Importantly, multivariate analysis showed that cytoplasmic expression of HuR in mesothelioma was a significant predictor of recurrence compared to existing prognostic factors such as depth of disease, presence of sarcomatous components, nuclear grade and necrosis. Notably, the hazard ratio was higher than that in the presence of a sarcomatous component. Because pathologists frequently disagree on whether a sarcomatous component exists [17], cytoplasmic HuR staining, which is simple to detect, may be a more useful biomarker for predicting recurrence.

The correlation of cHuR expression with prognosis in mesothelioma has been demonstrated previously [12], although it varies from the current study in various respects. First, although the previous report suggested that cytoplasmic expression of HuR lowers prognosis, this is the first to show that this is associated with the acquisition of chemotherapy resistance. Another major difference is that our study found no significant alteration in OS, whereas the previous study reported a significant difference in OS. This may be because this study examined OS over a longer period than the previous report. It has been reported that HuR translocates to the cytoplasm after chemotherapy [18], and it is possible that postoperative chemotherapy causes HuR to migrate to the cytoplasm of mesothelioma cells, and the difference owing to HuR cytoplasmic expression in the non‐chemotherapy stage may disappear in the long term. Notably, in our investigative study, OS was worse in the HuR cytoplasm‐positive group in the short term.

RNA‐Seq analysis showed that the cytoplasmic expression of HuR in mesothelioma cells resulted in changes in the mRNA levels of several genes. Among these, the 10 most up‐ and down‐regulated genes were examined (Tables S3 and S4). Five of the observed upregulated genes, *CALB2*, *LRRC15*, *IGFBP5*, *LY6K* and *TAGLN*, are reported to increase drug resistance [19, 20, 21, 22, 23, 24, 25], but none of the upregulated genes increased drug sensitivity. The binding of *CALB2* mRNA to HuR is also reported [19]. Among the down‐regulated genes, *ABCG1* and *EGR1* are reported to increase drug resistance, whereas *FA2H* is reported to increase drug sensitivity [26, 27, 28, 29, 30, 31]. Thus, although there was a mixture of alterations that increased drug resistance and sensitivity, the overall number of changes in genes that promoted drug resistance was considered dominant.

In GSEA, cytoplasmic expression of HuR promoted the E2F pathway, a gene cluster that primarily regulates the cell cycle and has been reported to contribute to drug resistance. However, the direct mechanism is not clear [32, 33, 34]. Suppression of the p53 pathway also contributes to drug resistance by decreasing apoptosis after chemotherapy‐induced DNA damage [35]. In summary, whereas cHuR expression in mesothelioma cell lines alters the expression levels of multiple genes, including up‐regulation of the E2F pathway and suppression of the p53 pathway, the overall expression pattern appears to shift in the direction of increased drug resistance. Compared with the RNA‐Seq results, western blot analysis showed that although the trend of change was maintained, the alteration at the protein level tended to be smaller than that in mRNA. The reason for this remains unclear, but this is consistent with the speculation that altering the expression of multiple genes, rather than a single or few genes, contributes to overall drug resistance.

The current findings indicate that inhibiting HuR function in the cytoplasm may be a way to overcome drug resistance in mesothelioma. Because of the large number of target genes, inhibiting HuR mRNA synthesis or inhibiting HuR cytoplasmic migration may be a viable option. As cHuR expression is not observed in non‐neoplastic mesothelial cells, the inhibition of cHuR transfer may be more effective in mesothelioma cells and a potential therapeutic approach. However, this study did not identify the mechanism by which HuR is translocated to the cytoplasm of mesothelial cells, and this requires further investigation for therapeutic application.

## Author Contributions

**Susumu Kirimura:** conceptualisation, data curation, formal analysis, funding acquisition, investigation, project administration, visualisation and writing the original draft. **Morito Kurata:** methodology, writing – review and editing, and supervision. **Hironori Ishibashi:** resources. **Yusuke Taniguchi:** investigation. **Yuko Kinowaki:** formal analysis. **Keisuke Sugita:** formal analysis. **Kenichi Okubo:** resources.

## Ethics Statement

As this was an observational study, informed consent was not obtained from the individual patients. Instead, the information was disclosed on the website of the Bioethics Research Centre at TMDU. This study was approved by the Ethics Committee of Tokyo Medical and Dental University (M2017‐122).

## Conflicts of Interest

The authors declare no conflicts of interest.

## Supporting information


**Figure S1.** Schematic representation of lentiviral vector constructs for NES‐HuRvector (top) and NLS‐HuR vector (bottom). HuR, Human antigen R; NES, nuclear export signal; NLS, nuclear localization signal; LTR, long terminal repeat; PGK/pgr, phosphoglycerate kinase promoter; Puro, puromycin resistance gene.

**Figure S2.** Western blotting analysis for E2F1 (a) and TP53 (b). No significant changes at the protein level were observed for E2F1 and TP53 itself by forced expression of HuR (c).

**Table S1.** Gene sets enriched in NES‐HuR transgenic cells.
**Table S2.** Gene sets suppressed in NES‐HuR transgenic cells.
**Table S3.** The 10 most up‐regulated genes in NES‐HuR transgenic cells.
**Table S4.** The 10 most down‐regulated genes in NES‐HuR transgenic cells.
**Table S5.** E2F family genes and TP53 expression in NES‐HuR transgenic cells.

## Data Availability

The datasets generated and analysed in the current study are available from the corresponding author upon reasonable request.
