# Supplementary figures and images for: Cytoplasmic HuR Expression Enhances Chemoresistance in Pleural Mesothelioma Through Increased Expression of CALB2, Promotion of the E2F Pathway, and Suppression of the p53 Pathway
Source: Thorac Cancer. 2025 Apr 9;16(7):e70062. doi: 10.1111/1759-7714.70062 (PMC11979354; doi:10.1111/1759-7714.70062)

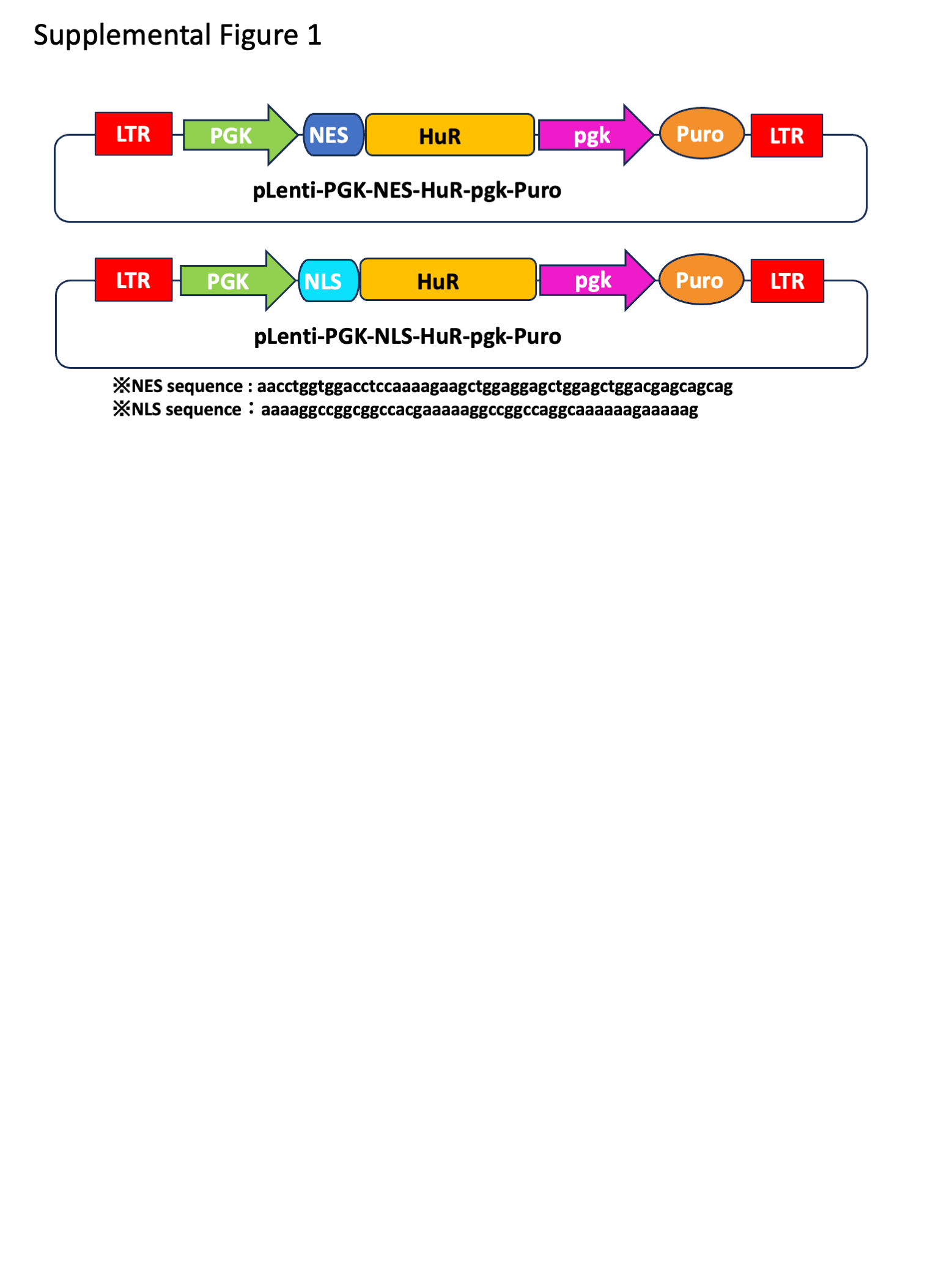

Supplement: Supplementary file 1 — Figure S1. Schematic representation of lentiviral vector constructs for NES‐HuRvector (top) and NLS‐HuR vector (bottom). HuR, Human antigen R; NES, nuclear export signal; NLS, nuclear localization signal; LTR, long terminal repeat; PGK/pgr, phosphoglycerate kinase promoter; Puro, puromycin resistance gene. [file TCA-16-e70062-s002.tiff]

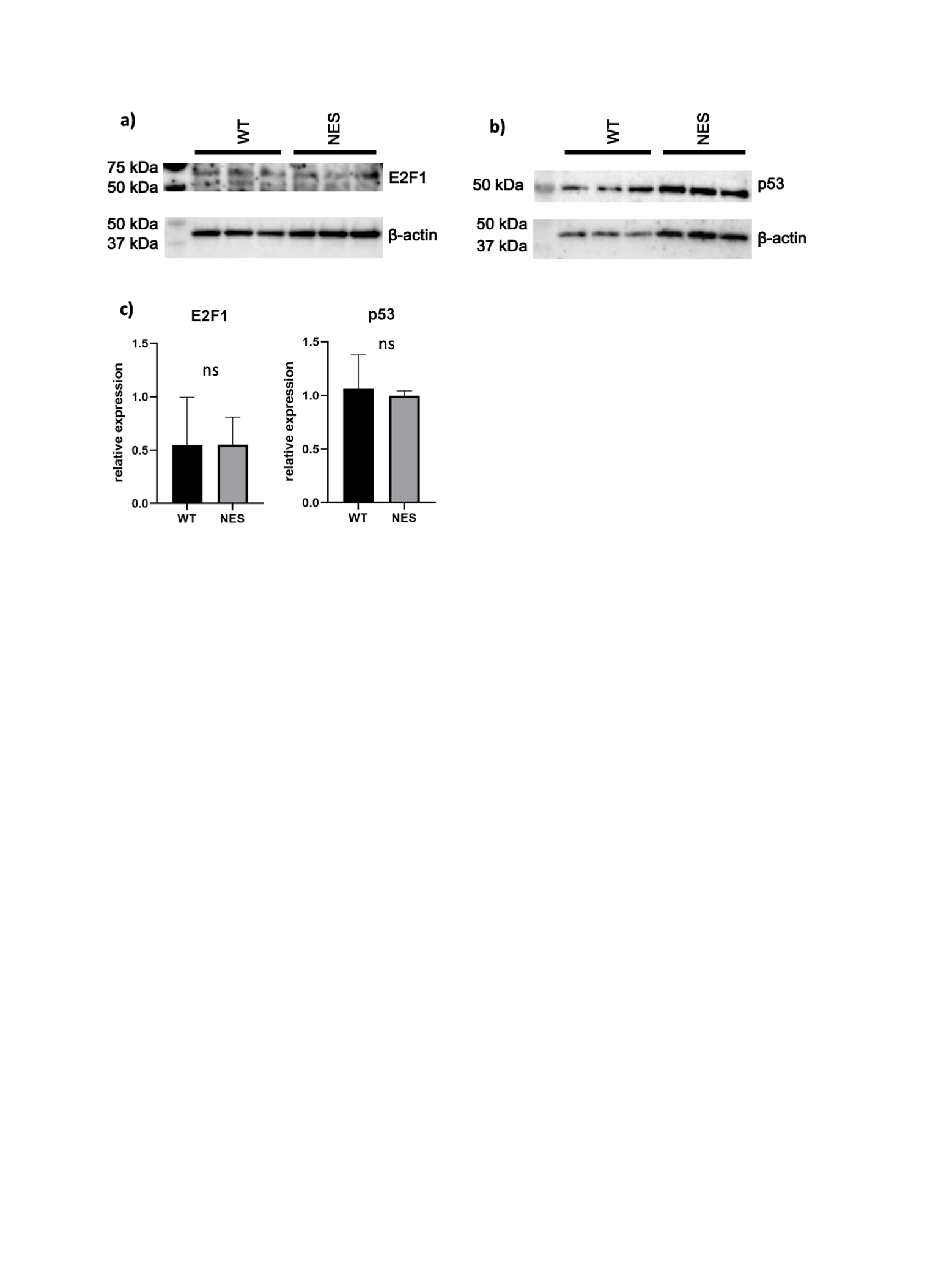

Supplement: Supplementary file 2 — Figure S2. Western blotting analysis for E2F1 (a) and TP53 (b). No significant changes at the protein level were observed for E2F1 and TP53 itself by forced expression of HuR (c). [file TCA-16-e70062-s003.tiff]
