# Supplementary material for: Cytoplasmic HuR Expression Enhances Chemoresistance in Pleural Mesothelioma Through Increased Expression of CALB2, Promotion of the E2F Pathway, and Suppression of the p53 Pathway
Source: Thorac Cancer. 2025 Apr 9;16(7):e70062. doi: 10.1111/1759-7714.70062 (PMC11979354; doi:10.1111/1759-7714.70062)
Supplement: Supplementary file 3 — Table S1. Gene sets enriched in NES‐HuR transgenic cells. Table S2. Gene sets suppressed in NES‐HuR transgenic cells. Table S3. The 10 most up‐regulated genes in NES‐HuR transgenic cells. Table S4. The 10 most down‐regulated genes in NES‐HuR transgenic cells. Table S5. E2F family genes and TP53 expression in NES‐HuR transgenic cells. [file TCA-16-e70062-s001.docx]

| **Gene sets** | **SIZE** | **ES** | **NES** | **NOM p-val** | **FDR q-val** | **FWER p-val** | **RANK AT MAX** | **LEADING EDGE** |
| --- | --- | --- | --- | --- | --- | --- | --- | --- |
| HALLMARK_E2F_TARGETS | 196 | 0.71 | 1.2 | 0 | 0.248 | 0.694 | 5618 | tags=78%, list=21%, signal=98% |
| HALLMARK_MYC_TARGETS_V1 | 195 | 0.61 | 1.22 | 0 | 0.282 | 0.694 | 7102 | tags=67%, list=27%, signal=91% |
| HALLMARK_G2M_CHECKPOINT | 195 | 0.68 | 1.25 | 0 | 0.283 | 0.694 | 5106 | tags=66%, list=19%, signal=81% |
| HALLMARK_MITOTIC_SPINDLE | 198 | 0.37 | 1.27 | 0 | 0.3 | 0.654 | 5798 | tags=34%, list=22%, signal=44% |
| HALLMARK_EPITHELIAL_MESENCHYMAL_TRANSITION | 189 | 0.28 | 1.37 | 0 | 0.302 | 0.567 | 2005 | tags=16%, list=8%, signal=17% |
| HALLMARK_GLYCOLYSIS | 189 | 0.23 | 1.46 | 0 | 0.322 | 0.362 | 2750 | tags=19%, list=10%, signal=21% |
| HALLMARK_MYC_TARGETS_V2 | 57 | 0.65 | 1.28 | 0 | 0.349 | 0.654 | 5710 | tags=72%, list=22%, signal=92% |
| HALLMARK_MTORC1_SIGNALING | 196 | 0.24 | 1.53 | 0 | 0.389 | 0.299 | 5343 | tags=28%, list=20%, signal=35% |

**Supplemental Table 1. Gene sets enriched in NES-HuR transgenic cells**

| **Gene sets** | **SIZE** | **ES** | **NES** | **NOM p-val** | **FDR q-val** | **FWER p-val** | **RANK AT MAX** | **LEADING EDGE** |
| --- | --- | --- | --- | --- | --- | --- | --- | --- |
| HALLMARK_WNT_BETA_CATENIN_SIGNALING | 39 | -0.51 | -1.88 | 0 | 0.063 | 0 | 1631 | tags=18%, list=6%, signal=19% |
| HALLMARK_ALLOGRAFT_REJECTION | 147 | -0.44 | -1.63 | 0 | 0.088 | 0.103 | 4102 | tags=26%, list=16%, signal=30% |
| HALLMARK_CHOLESTEROL_HOMEOSTASIS | 71 | -0.49 | -1.6 | 0 | 0.195 | 0.321 | 4394 | tags=31%, list=17%, signal=37% |
| HALLMARK_HYPOXIA | 191 | -0.32 | -1.59 | 0 | 0.162 | 0.321 | 4487 | tags=26%, list=17%, signal=31% |
| HALLMARK_ESTROGEN_RESPONSE_LATE | 181 | -0.4 | -1.59 | 0 | 0.142 | 0.321 | 4139 | tags=28%, list=16%, signal=33% |
| HALLMARK_UV_RESPONSE_UP | 150 | -0.3 | -1.58 | 0 | 0.129 | 0.321 | 4801 | tags=22%, list=18%, signal=27% |
| HALLMARK_INFLAMMATORY_RESPONSE | 155 | -0.55 | -1.58 | 0 | 0.12 | 0.321 | 4186 | tags=38%, list=16%, signal=45% |
| HALLMARK_ESTROGEN_RESPONSE_EARLY | 188 | -0.4 | -1.57 | 0 | 0.113 | 0.321 | 4519 | tags=29%, list=17%, signal=34% |
| HALLMARK_TNFA_SIGNALING_VIA_NFKB | 192 | -0.55 | -1.56 | 0 | 0.107 | 0.321 | 3904 | tags=36%, list=15%, signal=42% |
| HALLMARK_ANGIOGENESIS | 31 | -0.56 | -1.56 | 0 | 0.103 | 0.321 | 3480 | tags=39%, list=13%, signal=45% |
| HALLMARK_COMPLEMENT | 177 | -0.49 | -1.54 | 0 | 0.099 | 0.321 | 3308 | tags=29%, list=13%, signal=33% |
| HALLMARK_P53_PATHWAY | 189 | -0.37 | -1.53 | 0 | 0.1 | 0.321 | 4519 | tags=29%, list=17%, signal=35% |
| HALLMARK_XENOBIOTIC_METABOLISM | 172 | -0.41 | -1.52 | 0 | 0.097 | 0.321 | 5245 | tags=28%, list=20%, signal=35% |
| HALLMARK_APICAL_SURFACE | 40 | -0.46 | -1.51 | 0 | 0.095 | 0.321 | 5093 | tags=30%, list=19%, signal=37% |
| HALLMARK_INTERFERON_GAMMA_RESPONSE | 185 | -0.7 | -1.51 | 0 | 0.093 | 0.321 | 3999 | tags=50%, list=15%, signal=58% |
| HALLMARK_NOTCH_SIGNALING | 32 | -0.46 | -1.51 | 0 | 0.091 | 0.321 | 5040 | tags=31%, list=19%, signal=39% |
| HALLMARK_KRAS_SIGNALING_UP | 169 | -0.44 | -1.5 | 0 | 0.089 | 0.321 | 3504 | tags=28%, list=13%, signal=32% |
| HALLMARK_IL2_STAT5_SIGNALING | 175 | -0.41 | -1.47 | 0 | 0.093 | 0.371 | 5096 | tags=33%, list=19%, signal=40% |
| HALLMARK_COAGULATION | 114 | -0.45 | -1.47 | 0 | 0.092 | 0.371 | 5294 | tags=37%, list=20%, signal=46% |
| HALLMARK_BILE_ACID_METABOLISM | 94 | -0.45 | -1.46 | 0 | 0.09 | 0.371 | 7715 | tags=47%, list=29%, signal=66% |
| HALLMARK_IL6_JAK_STAT3_SIGNALING | 73 | -0.52 | -1.46 | 0 | 0.089 | 0.371 | 3669 | tags=37%, list=14%, signal=43% |
| HALLMARK_FATTY_ACID_METABOLISM | 140 | -0.38 | -1.45 | 0 | 0.091 | 0.371 | 5505 | tags=31%, list=21%, signal=39% |
| HALLMARK_APOPTOSIS | 153 | -0.45 | -1.45 | 0 | 0.089 | 0.371 | 4449 | tags=30%, list=17%, signal=36% |
| HALLMARK_HEME_METABOLISM | 176 | -0.29 | -1.38 | 0 | 0.122 | 0.545 | 8672 | tags=40%, list=33%, signal=59% |
| HALLMARK_MYOGENESIS | 180 | -0.32 | -1.32 | 0 | 0.136 | 0.595 | 4270 | tags=28%, list=16%, signal=33% |
| HALLMARK_INTERFERON_ALPHA_RESPONSE | 94 | -0.77 | -1.31 | 0 | 0.133 | 0.595 | 3999 | tags=67%, list=15%, signal=79% |
| HALLMARK_TGF_BETA_SIGNALING | 54 | -0.23 | -1.31 | 0 | 0.131 | 0.595 | 4577 | tags=15%, list=17%, signal=18% |
| HALLMARK_KRAS_SIGNALING_DN | 149 | -0.38 | -1.24 | 0 | 0.173 | 0.701 | 6265 | tags=40%, list=24%, signal=53% |

**Supplemental Table 2. Gene sets suppressed in NES-HuR transgenic cells**

**Supplemental Table 3. The 10 most up-regulated genes in NES-HuR transgenic cells**

| **Gene name** | **log2 Fold Change** | **P-value** | **Padj-value** |
| --- | --- | --- | --- |
| ACTG2 | 3.567187206 | 0.000176318 | 0.000947286 |
| CALB2 | 3.1002274959418 | 8.93547825902672e-38 | 9.94182857642633e-36 |
| AP000892.3 | 2.74009379656095 | 0.000432574144629611 | 0.0020945133274535 |
| LRRC15 | 2.62503425166633 | 2.31363177321581e-16 | 6.55167062422558e-15 |
| IGFBP5 | 2.3866163621517 | 0.0000024676183175153 | 0.0000193656809230515 |
| LY6K | 2.26763016372205 | 0.0000435169958174357 | 0.000268354807540854 |
| ACTL8 | 2.04161251889982 | 5.72248707760733e-10 | 7.95167203485418e-9 |
| SPTB | 1.89258865655113 | 0.000252103939452996 | 0.00130528270697644 |
| TAGLN | 1.78520217802887 | 7.92980937059784e-121 | 1.55503561757424e-117 |
| AP000688.2 | 1.74498792145178 | 0.0032575252986696 | 0.0124552904912329 |

**Supplemental Table 4. The 10 most down-regulated genes in NES-HuR transgenic cells**

| **Gene name** | **log2 Fold Change** | **P-value** | **Padj-value** |
| --- | --- | --- | --- |
| ABCG1 | -5.34565553384157 | 0.000070185535907002 | 0.000411154102803976 |
| EGR1 | -3.21896155643923 | 5.64094047835314e-39 | 6.65376497927851e-37 |
| NPNT | -3.1995037655813 | 3.18654309419323e-9 | 3.98648230157124e-8 |
| ZNF804A | -3.11455304514683 | 0.00037796977487616 | 0.00185822307372523 |
| RSAD2 | -3.04126570638816 | 2.94013223425613e-211 | 2.30623972455051e-207 |
| RAET1L | -2.85731375005589 | 0.000533687939815658 | 0.00251955955456757 |
| PI16 | -2.78721731817793 | 0.000144625917185689 | 0.000793014887608262 |
| IFITM1 | -2.76535818586596 | 8.42943446813494e-18 | 2.72100757070166e-16 |
| CSAG3 | -2.75562209829722 | 0.0000338990868163944 | 0.00021344936625189 |
| FA2H | -2.73682773526128 | 2.58008109362213e-8 | 2.80890438561721e-7 |

**Supplemental Table 5. E2F family genes and TP53 expression in NES-HuR transgenic cells**

| **Gene name** | **log2 Fold Change** | **P-value** | **Padj-value** |
| --- | --- | --- | --- |
| E2F1 | 0.511720940704328 | 1.74052412314415e-16 | 4.99183591295893e-15 |
| E2F2 | 0.346691490507694 | 0.000356951277343377 | 0.0017659576281813 |
| E2F3 | 0.319642223475605 | 0.0000112071525708908 | 0.00007743279068364 |
| E2F3P2 | -1.7305092534811 | 0.623085566865275 | 1 |
| E2F4 | 0.093289669574037 | 0.174610109042651 | 0.325098907033124 |
| E2F5 | -0.428786465488417 | 0.00727774691117941 | 0.0248202812049093 |
| E2F6 | -0.007604472457609 | 0.943113156242426 | 0.969814597955243 |
| E2F6P1 | 0.214504834586004 | 0.952319093640779 | 1 |
| E2F7 | 0.733869917607062 | 2.53445737982282e-11 | 4.18092191111046e-10 |
| E2F8 | 0.529403768925395 | 0.00000120792472825966 | 4.18092191111046e-10 |
| TP53 | -0.449666298981676 | 0.101754806501731 | 0.215341886469602 |
